# 2-Amino-3-nitro­benzoic acid

**DOI:** 10.1107/S1600536812002036

**Published:** 2012-01-21

**Authors:** Yip-Foo Win, Chen-Shang Choong, Siang-Guan Teoh, Ching Kheng Quah, Hoong-Kun Fun

**Affiliations:** a Department of Chemical Science, Faculty of Science, Universiti Tunku Abdul Rahman, Perak Campus, Jalan Universiti, Bandar Barat, 31900 Kampar, Perak, Malaysia; b School of Chemical Sciences, Universiti Sains Malaysia, 11800 USM, Penang, Malaysia; c X-ray Crystallography Unit, School of Physics, Universiti Sains Malaysia, 11800 USM, Penang, Malaysia

## Abstract

The title compound, C_7_H_6_N_2_O_4_, is approximately planar (r.m.s. deviation = 0.026 Å for the 13 non-H atoms). The mol­ecular structure is stabilized by intra­molecular N—H⋯O hydrogen bonds, which generate *S*(6) ring motifs. In the crystal, mol­ecules are linked *via* inter­molecular N—H⋯O, O—H⋯O and C—H⋯O hydrogen bonds into a three-dimensional network.

## Related literature

For general background to the title compound and related structures, see: Win *et al.* (2010 ▶, 2011*a* ▶,*b* ▶,*c* ▶). For standard bond-length data, see: Allen *et al.* (1987 ▶). For hydrogen-bond motifs, see: Bernstein *et al.* (1995 ▶). For the stability of the temperature controller used in the the data collection, see: Cosier & Glazer (1986 ▶).
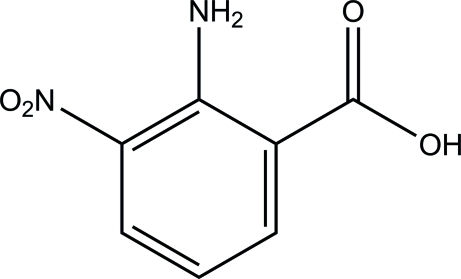


## Experimental

### 

#### Crystal data

C_7_H_6_N_2_O_4_
*M* *_r_* = 182.14
Monoclinic, 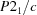

*a* = 9.0231 (3) Å
*b* = 7.4338 (2) Å
*c* = 11.0392 (4) Å
β = 92.114 (1)°
*V* = 739.96 (4) Å^3^
*Z* = 4
Mo *K*α radiation
μ = 0.14 mm^−1^
*T* = 100 K
0.34 × 0.26 × 0.16 mm

#### Data collection

Bruker SMART APEXII CCD area-detector diffractometer
Absorption correction: multi-scan (*SADABS*; Bruker, 2009 ▶) *T*_min_ = 0.956, *T*_max_ = 0.979
22297 measured reflections
3247 independent reflections
2707 reflections with *I* > 2σ(*I*)
*R*_int_ = 0.030

#### Refinement

*R*[*F* ^2^ > 2σ(*F* ^2^)] = 0.042
*wR*(*F* ^2^) = 0.121
*S* = 1.04
3247 reflections
130 parameters
H atoms treated by a mixture of independent and constrained refinement
Δρ_max_ = 0.55 e Å^−3^
Δρ_min_ = −0.33 e Å^−3^

### 

Data collection: *APEX2* (Bruker, 2009 ▶); cell refinement: *SAINT* (Bruker, 2009 ▶); data reduction: *SAINT*; program(s) used to solve structure: *SHELXTL* (Sheldrick, 2008 ▶); program(s) used to refine structure: *SHELXTL*; molecular graphics: *SHELXTL*; software used to prepare material for publication: *SHELXTL* and *PLATON* (Spek, 2009 ▶).

## Supplementary Material

Crystal structure: contains datablock(s) global, I. DOI: 10.1107/S1600536812002036/qm2050sup1.cif

Structure factors: contains datablock(s) I. DOI: 10.1107/S1600536812002036/qm2050Isup2.hkl

Supplementary material file. DOI: 10.1107/S1600536812002036/qm2050Isup3.cml

Additional supplementary materials:  crystallographic information; 3D view; checkCIF report

## Figures and Tables

**Table 1:** Hydrogen-bond geometry (Å, °)

*D*—H⋯*A*	*D*—H	H⋯*A*	*D*⋯*A*	*D*—H⋯*A*
N2—H1*N*2⋯O2	0.892 (17)	1.958 (16)	2.6082 (11)	128.5 (13)
N2—H1*N*2⋯O1^i^	0.892 (17)	2.499 (17)	3.2885 (12)	147.8 (14)
N2—H2*N*2⋯O3	0.872 (15)	1.982 (16)	2.6582 (10)	133.4 (14)
O4—H1*O*4⋯O3^ii^	0.83 (2)	1.81 (2)	2.6397 (10)	176.2 (17)
C3—H3*A*⋯O1^iii^	0.95	2.49	3.4349 (12)	176

Symmetry codes: (i) 
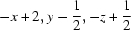
; (ii) 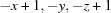; (iii) 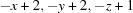.

**Fig. 1.**
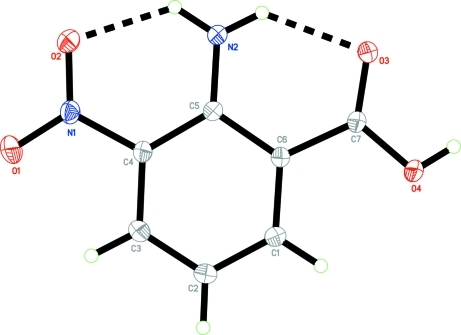
The molecular structure of the title compound showing 50% probability displacement ellipsoids for non-H atoms. Intramolecular hydrogen bonds are shown as dashed lines.

**Fig. 2.**
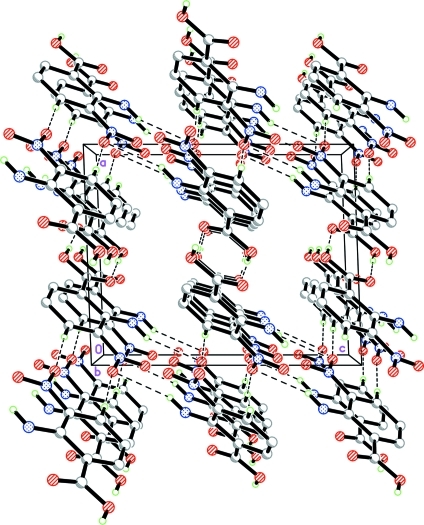
The crystal structure of the title compound, viewed along the b axis. H atoms not involved in hydrogen bonds (dashed lines) have been omitted for clarity.

## supplementary crystallographic information

### Comment

In the study of organotin(IV) carboxylate complexes, 2-amino-5-nitrobenzoic, 2-amino-3-nitrobenzoic, 4-amino-3-nitrobenzoic and 5-amino-2-nitrobenzoic acids have been utilized in the synthesis work (Win *et al.*, 2010, 2011*a*, 2011*b*, 2011*c*). The carboxylate anions of the acids are found to be bonded to tin(IV) atom moieties in both a monodentate and a bidentate manner resulting in structural diversity for organotin(IV) carboxylate complexes (Win *et al.*, 2010, 2011*a*, 2011*b*, 2011*c*).

The title compound, Fig.1, is approximately planar (r.m.s. deviation = 0.026 Å for the 13 non-H atoms). Bond lengths (Allen *et al.*, 1987) and angles are within normal ranges and are comparable to related structures (Win *et al.*, 2010, 2011*a*, 2011*b*, 2011*c*). The molecular structure is stabilized by intramolecular N2–H1N2···O2 and N2–H2N2···O3 hydrogen bonds (Table 1), which generate *S*(6) ring motifs (Fig. 1, Bernstein *et al.*, 1995).

In the crystal structure, Fig. 2, molecules are linked *via* intermolecular N2–H1N2···O1, O4–H1O4···O3 and C3–H3A···O1 hydrogen bonds (Table 1) into a three-dimensional network.

### Experimental

The attempt to prepare organotin(IV) carboxylate complexes by heating under reflux the mixture of 2-amino-3-nitrobenzoic acid (2 mmol) and dimethyltin(IV) oxide (4 mmol) for 4 h in 50 ml of methanol was unsuccessful. The resulting orange solution was filtered and orange crystals were obtained after 2 weeks. Unfortunately, the crystals obtained were found to be the starting material (2-amino-3-nitrobenzoic acid) with the melting point of 482 K.

### Refinement

Atoms H1O4, H1N2 and H2N2 were located in a difference Fourier map and refined freely with O-H = 0.83 (2) Å and N-H = 0.875 (18) and 0.893 (17) Å. The remaining H atoms were positioned geometrically and refined using a riding model with C–H = 0.95 Å and *U*_iso_(H) = 1.2 *U*_eq_(C).

### Crystal data

**Table:**

C_7_H_6_N_2_O_4_	*F*(000) = 376
*M**_r_* = 182.14	*D*_x_ = 1.635 Mg m^−^^3^
Monoclinic, *P*2_1_/*c*	Mo *K*α radiation, λ = 0.71073 Å
Hall symbol: -P 2ybc	Cell parameters from 9956 reflections
*a* = 9.0231 (3) Å	θ = 2.9–34.8°
*b* = 7.4338 (2) Å	µ = 0.14 mm^−^^1^
*c* = 11.0392 (4) Å	*T* = 100 K
β = 92.114 (1)°	Block, orange
*V* = 739.96 (4) Å^3^	0.34 × 0.26 × 0.16 mm
*Z* = 4	

### Data collection

**Table:**

Bruker SMART APEXII CCD area-detector diffractometer	3247 independent reflections
Radiation source: fine-focus sealed tube	2707 reflections with *I* > 2σ(*I*)
graphite	*R*_int_ = 0.030
φ and ω scans	θ_max_ = 35.0°, θ_min_ = 2.3°
Absorption correction: multi-scan (*SADABS*; Bruker, 2009)	*h* = −14→12
*T*_min_ = 0.956, *T*_max_ = 0.979	*k* = −11→11
22297 measured reflections	*l* = −17→17

### Refinement

**Table:**

Refinement on *F*^2^	Primary atom site location: structure-invariant direct methods
Least-squares matrix: full	Secondary atom site location: difference Fourier map
*R*[*F*^2^ > 2σ(*F*^2^)] = 0.042	Hydrogen site location: inferred from neighbouring sites
*wR*(*F*^2^) = 0.121	H atoms treated by a mixture of independent and constrained refinement
*S* = 1.04	*w* = 1/[σ^2^(*F*_o_^2^) + (0.0662*P*)^2^ + 0.1903*P*] where *P* = (*F*_o_^2^ + 2*F*_c_^2^)/3
3247 reflections	(Δ/σ)_max_ = 0.001
130 parameters	Δρ_max_ = 0.55 e Å^−^^3^
0 restraints	Δρ_min_ = −0.33 e Å^−^^3^

### Special details

**Table:**

Experimental. The crystal was placed in the cold stream of an Oxford Cryosystems Cobra open-flow nitrogen cryostat (Cosier & Glazer, 1986) operating at 100.0 (1) K.
Geometry. All esds (except the esd in the dihedral angle between two l.s. planes) are estimated using the full covariance matrix. The cell esds are taken into account individually in the estimation of esds in distances, angles and torsion angles; correlations between esds in cell parameters are only used when they are defined by crystal symmetry. An approximate (isotropic) treatment of cell esds is used for estimating esds involving l.s. planes.
Refinement. Refinement of F^2^ against ALL reflections. The weighted R-factor wR and goodness of fit S are based on F^2^, conventional R-factors R are based on F, with F set to zero for negative F^2^. The threshold expression of F^2^ > 2sigma(F^2^) is used only for calculating R-factors(gt) etc. and is not relevant to the choice of reflections for refinement. R-factors based on F^2^ are statistically about twice as large as those based on F, and R- factors based on ALL data will be even larger.

### Fractional atomic coordinates and isotropic or equivalent isotropic displacement parameters (Å^2^)

**Table:**

	*x*	*y*	*z*	*U*_iso_*/*U*_eq_	
O1	1.00882 (10)	0.85440 (11)	0.40698 (8)	0.03096 (19)	
O2	0.99701 (8)	0.61460 (10)	0.29827 (6)	0.02030 (14)	
O3	0.62645 (7)	0.11784 (9)	0.43650 (6)	0.01909 (14)	
O4	0.51199 (8)	0.18983 (10)	0.60595 (6)	0.01981 (14)	
N1	0.95660 (8)	0.70510 (10)	0.38522 (6)	0.01587 (14)	
N2	0.81247 (9)	0.35687 (11)	0.34730 (7)	0.01954 (16)	
C1	0.64687 (9)	0.51128 (12)	0.62950 (7)	0.01552 (15)	
H1A	0.5784	0.4697	0.6865	0.019*	
C2	0.71159 (10)	0.67982 (12)	0.64617 (8)	0.01749 (16)	
H2A	0.6870	0.7528	0.7131	0.021*	
C3	0.81229 (9)	0.73922 (12)	0.56356 (7)	0.01620 (15)	
H3A	0.8579	0.8536	0.5742	0.019*	
C4	0.84738 (9)	0.63239 (11)	0.46482 (7)	0.01390 (14)	
C5	0.78217 (8)	0.45981 (11)	0.44292 (7)	0.01334 (14)	
C6	0.67970 (9)	0.40152 (11)	0.53140 (7)	0.01354 (14)	
C7	0.60572 (9)	0.22530 (11)	0.51976 (7)	0.01458 (15)	
H1N2	0.8797 (19)	0.393 (2)	0.2953 (15)	0.034 (4)*	
H2N2	0.7714 (18)	0.251 (2)	0.3400 (14)	0.033 (4)*	
H1O4	0.471 (2)	0.092 (3)	0.5901 (16)	0.044 (5)*	

### Atomic displacement parameters (Å^2^)

**Table:**

	*U*^11^	*U*^22^	*U*^33^	*U*^12^	*U*^13^	*U*^23^
O1	0.0415 (4)	0.0181 (3)	0.0343 (4)	−0.0153 (3)	0.0144 (3)	−0.0043 (3)
O2	0.0229 (3)	0.0216 (3)	0.0168 (3)	−0.0046 (2)	0.0065 (2)	−0.0007 (2)
O3	0.0214 (3)	0.0154 (3)	0.0210 (3)	−0.0059 (2)	0.0076 (2)	−0.0043 (2)
O4	0.0231 (3)	0.0189 (3)	0.0180 (3)	−0.0087 (2)	0.0080 (2)	−0.0028 (2)
N1	0.0173 (3)	0.0151 (3)	0.0153 (3)	−0.0030 (2)	0.0017 (2)	0.0023 (2)
N2	0.0221 (3)	0.0175 (3)	0.0197 (3)	−0.0068 (3)	0.0094 (3)	−0.0058 (3)
C1	0.0162 (3)	0.0166 (3)	0.0139 (3)	−0.0015 (3)	0.0025 (2)	−0.0015 (3)
C2	0.0198 (3)	0.0165 (4)	0.0163 (3)	−0.0011 (3)	0.0029 (3)	−0.0036 (3)
C3	0.0179 (3)	0.0138 (3)	0.0169 (3)	−0.0013 (3)	0.0008 (3)	−0.0014 (3)
C4	0.0143 (3)	0.0133 (3)	0.0142 (3)	−0.0016 (2)	0.0016 (2)	0.0009 (2)
C5	0.0135 (3)	0.0134 (3)	0.0132 (3)	−0.0007 (2)	0.0019 (2)	−0.0003 (2)
C6	0.0135 (3)	0.0128 (3)	0.0144 (3)	−0.0016 (2)	0.0021 (2)	−0.0007 (2)
C7	0.0143 (3)	0.0145 (3)	0.0151 (3)	−0.0025 (3)	0.0024 (2)	0.0003 (3)

### Geometric parameters (Å, °)

**Table:**

O1—N1	1.2260 (10)	C1—C6	1.3964 (11)
O2—N1	1.2380 (10)	C1—H1A	0.9500
O3—C7	1.2371 (10)	C2—C3	1.3832 (12)
O4—C7	1.3227 (10)	C2—H2A	0.9500
O4—H1O4	0.83 (2)	C3—C4	1.3945 (11)
N1—C4	1.4490 (10)	C3—H3A	0.9500
N2—C5	1.3401 (11)	C4—C5	1.4283 (11)
N2—H1N2	0.893 (17)	C5—C6	1.4364 (11)
N2—H2N2	0.875 (18)	C6—C7	1.4737 (11)
C1—C2	1.3916 (12)		
			
C7—O4—H1O4	108.3 (12)	C2—C3—H3A	119.8
O1—N1—O2	121.45 (7)	C4—C3—H3A	119.8
O1—N1—C4	118.94 (7)	C3—C4—C5	122.67 (7)
O2—N1—C4	119.59 (7)	C3—C4—N1	116.15 (7)
C5—N2—H1N2	119.8 (11)	C5—C4—N1	121.17 (7)
C5—N2—H2N2	119.3 (10)	N2—C5—C4	123.47 (7)
H1N2—N2—H2N2	120.7 (15)	N2—C5—C6	121.24 (7)
C2—C1—C6	121.91 (7)	C4—C5—C6	115.30 (7)
C2—C1—H1A	119.0	C1—C6—C5	120.75 (7)
C6—C1—H1A	119.0	C1—C6—C7	118.60 (7)
C3—C2—C1	118.87 (8)	C5—C6—C7	120.64 (7)
C3—C2—H2A	120.6	O3—C7—O4	121.60 (8)
C1—C2—H2A	120.6	O3—C7—C6	123.94 (7)
C2—C3—C4	120.47 (8)	O4—C7—C6	114.46 (7)
			
C6—C1—C2—C3	−0.58 (13)	N1—C4—C5—C6	177.80 (7)
C1—C2—C3—C4	0.47 (13)	C2—C1—C6—C5	−0.28 (13)
C2—C3—C4—C5	0.49 (13)	C2—C1—C6—C7	−179.61 (8)
C2—C3—C4—N1	−178.63 (8)	N2—C5—C6—C1	−178.81 (8)
O1—N1—C4—C3	−1.42 (12)	C4—C5—C6—C1	1.16 (11)
O2—N1—C4—C3	177.38 (7)	N2—C5—C6—C7	0.50 (13)
O1—N1—C4—C5	179.46 (8)	C4—C5—C6—C7	−179.53 (7)
O2—N1—C4—C5	−1.75 (12)	C1—C6—C7—O3	179.89 (8)
C3—C4—C5—N2	178.69 (8)	C5—C6—C7—O3	0.55 (13)
N1—C4—C5—N2	−2.24 (13)	C1—C6—C7—O4	0.60 (11)
C3—C4—C5—C6	−1.28 (12)	C5—C6—C7—O4	−178.73 (7)

### Hydrogen-bond geometry (Å, °)

**Table:**

*D*—H···*A*	*D*—H	H···*A*	*D*···*A*	*D*—H···*A*
N2—H1N2···O2	0.892 (17)	1.958 (16)	2.6082 (11)	128.5 (13)
N2—H1N2···O1^i^	0.892 (17)	2.499 (17)	3.2885 (12)	147.8 (14)
N2—H2N2···O3	0.872 (15)	1.982 (16)	2.6582 (10)	133.4 (14)
O4—H1O4···O3^ii^	0.83 (2)	1.81 (2)	2.6397 (10)	176.2 (17)
C3—H3A···O1^iii^	0.95	2.49	3.4349 (12)	176

Symmetry codes: (i) −*x*+2, *y*−1/2, −*z*+1/2; (ii) −*x*+1, −*y*, −*z*+1; (iii) −*x*+2, −*y*+2, −*z*+1.
